# Lipidomic analyses reveal the dysregulation of oxidized fatty acids (OxFAs) and acyl-carnitines (CARs) in major depressive disorder: a case-control study

**DOI:** 10.1186/s12888-025-07191-7

**Published:** 2025-08-01

**Authors:** Lei He, Na Duan, Chong Wang, Ru Shan, Jing Li, Lin Wang, Qiuli Liu, Junwei Tao, Li Liu, Xiaoliang Ma, Bing Cao

**Affiliations:** 1 The Affiliated Brain Hospital of Zhengzhou University(The Second People’s Hospital of Zhumadian), Zhumadian, Henan 463000 P. R. China; 2 Key Laboratory of Cognition and Personality, Faculty of Psychology, Ministry of Education, Southwest University, Chongqing, P. R. China

**Keywords:** Acyl-carnitine, Fatty acid, Oxidized, Lipids, Major depressive disorder

## Abstract

**Background:**

Growing data indicated that individuals diagnosed with major depressive disorder (MDD) had widespread inflammation, oxidative stress, and metabolic dysregulation. The objective of our study was to detect alterations in lipids of individuals with MDD, with the ultimate aim of developing potential biomarkers that may contribute to the diagnosis and treatment of MDD.

**Methods:**

The current study was a single-center cross-sectional case-control design. Serum samples were obtained from 107 individuals diagnosed with MDD and 97 healthy controls (HC) aged 18 to 60 years. Lipidomics analysis was performed using an Ultimate 3000 UHPLC system coupled with a Q-Exactive HF MS platform. All data were processed using the specialized online software Metaboanalyst 5.0.

**Results:**

Based on the filtering criteria of false discovery rate (FDR) -adjusted *P* < 0.05, variable importance in projection (VIP) > 1.5, and fold change (FC) > 2.0 or < 0.5, a total of 40 lipids were identified as significantly different. In patients with MDD, we observed an increase in 11 types of oxidized fatty acids (OxFAs) and a decrease in 5 types of OxFAs. Additionally, we found that 8 types of acyl-carnitines (CARs) decreased, primarily comprising singular carbon chain structures, while 3 types of CARs increased, all with numerical carbon chain patterns. Lipid profiles yield a high area under the receiver operating characteristic (ROC) curve for differentiating MDD, with the most prominent ROC ranking being mostly attributed to OxFAs.

**Conclusions:**

Our research found significant variations in lipid levels, specifically increased OxFAs and decreased CARs, in individuals with MDD compared to HCs. Supplementation with PUFAs and acyl-carnitines warrants further investigation as a potential strategy for the management of MDD. Nevertheless, further investigation is necessary, and exercise prudence is required when examining and implementing their forms and proportions.

**Supplementary Information:**

The online version contains supplementary material available at 10.1186/s12888-025-07191-7.

## Introduction

Major depressive disorder (MDD) is a prevalent mental disorder characterized by persistent symptoms of depressed mood lasting for at least two weeks [1]. The disease burden of MDD is typically long-term and severe due to associated emotional stress, functional impairment, health issues, and suicide risk [2, 3]. The etiology of MDD remains poorly understood, and effective therapies are lacking. Currently, the primary intervention for MDD is around the administration of antidepressant medication. Nevertheless, the efficacy of the majority of antidepressants is not optimal, as approximately 50% of patients do not respond. Additionally, these medications fail to address relapse and treatment resistance [4, 5]. Identification of reliable biomarkers to understand the pathogenesis of MDD could substantially influence the development of novel therapies.

As the interest in precision medicine continues to increase, an increasing number of studies are investigating the correlations between specific biomarkers and MDD. Emerging evidence indicates that MDD may be associated with widespread dysregulation of multiple biological systems [6]. Researchers have proposed several biomarkers for the identification of MDD. A systematic review summarized 75 prospective studies examining biomarkers of MDD. The results revealed that biomarkers of MDD are associated with various factors, including neuroimaging (24 studies), gastrointestinal factors (1 study), immunology (8 studies), neurotrophic (2 studies), neurotransmitters (1 study), hormones (39 studies), and oxidative stress (1 study). The authors highlighted cortisol as a potential indicator for MDD [7]. Specific to the field of metabolomics, there have been some comparisons between individuals with MDD and healthy controls in previous studies. Liu et al. reported that levels of acyl-carnitines (CARs), ether lipids, and tryptophan decreased pronouncedly, whereas lysophosphatidylcholines (LPCs), lysophosphatidylethanolamines (LPEs), and phosphatidylethanolamines (PEs) increased in MDD subjects [8]. This study and a systematic review consistently reported abnormalities in kynurenine and acyl-carnitine levels in individuals with MDD [9]. Moreover, preclinical and clinical evidence suggests that ceramides, phosphatidylcholines (PCs), and sphingomyelins (SMs) may also contribute to the pathophysiology of MDD [10]. Brunkhorst-Kanaan et al. found that differences between MDD and bipolar disorder patients versus controls mainly originated from ceramides and their hexosyl-metabolites, and antidepressant use could increase ceramide levels [11]. The study from Knowles and colleagues found a shared genetic etiology between MDD and ether-phosphatidylcholine species [12]. Thus, phospho- and sphingolipid molecules can be considered as potential biomarkers of MDD, possibly related to the linoleic/arachidonic acid inflammation pathway [12, 13].

Above all, there have been no leading biological theories to propose changes to current diagnostic and therapeutic practices. To our knowledge, existing research has revealed only limited lipids-specific findings. Although previous studies have reported lipid-based predictive markers for mental disorders, such as schizophrenia, depression, and bipolar disorder [14], understanding of lipid metabolism in these disorders, especially MDD, remains inconsistent. Therefore, we conducted this lipidomic analysis to detect alterations in lipids in individuals with MDD, aiming to identify potential biomarkers that could contribute to the diagnosis and treatment of MDD.

##  Methods

### Ethical approval

The authors affirm that all procedures contributing to this work complied with the ethical standards of relevant national and institutional committees on human experimentation and with the Helsinki Declaration of 1975, as its 2008 revisions. The Medical Ethics Committee of Zhumadian Second People's Hospital in Henan Province approved all procedures involving human subjects/patients (Approval no. IRB-2021-006-02). Prior to their participation in the study, all subjects provided written informed consent.

### Study design and participants

This was a single-center cross-sectional case-control study, which consisted of 214 participants. Specifically, a total of 107 individuals diagnosed with MDD, ranging in age from 18 to 60 years, were recruited from Zhumadian Second People's Hospital in Henan, China, during the period from March 2022 to January 2023. Additionally, 97 volunteers were recruited from the health examination population in the physical examination center of the same hospital. All participants had no history of mental disorders (referred to as healthy controls or HCs) were also enlisted from the same area and time frame as the MDD group.

The inclusion criteria of MDD were as follows: (1) Trained psychiatrists confirmed the diagnosis of MDD based on the criteria outlined in the Diagnostic and Statistical Manual of Mental Disorders-5th version (DSM-V); current major depressive episode (MDE) was confirmed by the Mini International Neuropsychiatric Interview (M.I.N.I 5.0.); (2) The study included patients with MDD who were experiencing their first episode, or drug-treated but had not previously taken antidepressants or antipsychotics within the past month; (3) The minimum education level of the participants was primary school; (4) The severity of depression was assessed using the Hamilton Depression Scale (HAMD)- 24 version, with a score of 20 or higher indicating significant depression; (5) The study included individuals between the ages of 18 and 60, with no restrictions based on gender. The inclusion criteria for HCs were as follows: (1) Ensure matches with MDD groups in terms of gender, age, and place of residence, no history of DSM-defined mental illness, and a HAMD-24 score of less than 20; (2) Require a minimum educational level of primary school; (3) Confirm that standard medical tests such as hematology, urine analysis, feces analysis, liver function, fasting blood glucose, renal function, chest X-ray, and EKG show normal results.

The exclusion criteria of the two groups are the same: (1) Previous occurrence of organic brain disease or diagnosed neurologic disorder such as Parkinson's disease, cerebral hemorrhage, massive cerebral infarction, encephalitis, or epilepsy; (2) Severe medical conditions that are clinically significant or unstable, including liver, kidney, gastrointestinal, respiratory, cardiovascular, endocrine, blood, neurological, genitourinary, musculoskeletal, or metabolic-related diseases and problems; (3) Intellectual disabilities; (4) History of alcohol, drugs, chemicals, substances, or psychoactive substance abuse; (5) Impairment in vision or hearing; (6) Pregnant and lactating women.

### Demographic and clinical information collection 

Demographic information from all participants was collected by trained healthcare workers. Information was collected on gender, age, body mass index, education level, smoking and alcohol use. A smoker was defined as smoking at least one cigarette a day, and a drinker was defined as drinking at least once a week in the current study. The HAMD-24 was employed to quantify the severity of psychiatric symptoms.

### Sample preparation and detection

Approximately 8.5 ml of intravenous morning blood samples were taken from the forearm vein following a 12-hour fasting period in the morning (between 7-9 a.m.) for the purpose of isolating serum. The serum samples were centrifuged at 3000× g for 10 minutes at 4°C. The resulting serum was then divided into labeled 1.5-ml Eppendorf vials and stored at -80 °C until needed. The liquid-liquid extraction technique was used to extract lipids from the serum samples. Briefly, lipids were extracted from serum samples using protein precipitation as follows: A volume of 50 μL serum was extracted with 300 μL of cold methanol. The solution was agitated using a vortex mixer for 5 minutes at a speed of 1200 revolutions per minute (rpm) and thereafter placed in a storage environment with a temperature of -20 °C for 1 hour. Next, the mixture underwent centrifugation at 13,000×g for 20 min at a temperature of 4 °C. The supernatant was collected and evaporated at room temperature under vacuum. Equal volumes of the supernatant from healthy control samples were pooled to prepare quality control (QC) samples. The evaporated samples were stored at -80 °C until LC-MS analysis.

The untargeted lipidomics analysis was performed using an Ultimate 3000 UHPLC system coupled with Q-Exactive HF MS (Thermo Fisher Scientific, Waltham, MA, USA). The process of chromatographic separation was carried out using a phase reversed-phase X-select CSH C18 column (2.1 mm × 100 mm, 2.5 μm, Waters, USA) at 40 °C. The composition of mobile phase A was 5:95 acetonitrile: water (v/v) with 5 mM ammonium formate and 0.1% formic acid; mobile phase B, on the other hand, consisted of 95:5 acetonitrile: water (v/v) with 5 mM ammonium formate and 0.1% formic acid. The gradient was 0 min, 2% B; 1 min, 2% B; 18 min, 100% B; 22 min, 100% B, 22.1 min, 2% B; 26 min, 2% B. The column flow rate was 0.3 mL/min, autosampler temperature was 8 °C. The samples were suspended with 50 μL of acetonitrile: water (1:1, v/v) solution, and the injection volume was 10 μL. Data-dependent acquisition (DDA) based metabolomic data acquisition was performed using the Q-Exactive HF MS (Thermo Scientific). Acquisition was performed using positive-negative ion switching mode in TOP 10 mode. Samples were analyzed in random order. QC samples were analyzed 10 times at the start of the batch analysis and subsequently after every 10 samples.

### Data processing

Raw data obtained from the DDA-MS were processed on MS-DIAL software v3.6 according to the user guide as previously described [15]. Briefly, the raw MS data were converted into the standardized .abf file format using the Reifycs ABF converter (http://www.reifycs.com/AbfConverter/index.html). Next, MS-DIAL software was employed to perform feature detection, spectra deconvolution, metabolite identification, and peak alignment. MS1 and MS2 spectra-based lipid identification was conducted in MS-DIAL by searching the acquired spectra against the software’s LipidBlast-based in silico spectra database (version: LipidDBs- VS23- FiehnO), which includes information on common lipid species. The tolerance for MS1 and MS/MS search was set to 0.01 Da and 0.05 Da, respectively. The threshold for the identification score was established at 70%. The remaining parameters used in MS-DIAL were set as default.

### Statistical analysis

The analysis of the basic characteristics of the included participants was conducted by SPSS 28.0 (Statistical Package for Social Sciences). Continuous variables were summarized using either the mean and standard deviation (SD) or median and interquartile range (IQR), and categorical variables were summarized using frequencies and proportions (N, %). The independent samples t-test was employed to compare differences between two groups for continuous variables that followed a normal distribution, while the Mann-Whitney U test was utilized to compare differences between two groups for continuous variables that did not adhere to a normal distribution. The chi-square test was employed to compare the frequency distribution of categorical data between the two groups.

The online software Metaboanalyst 5.0 (https://www.metaboanalyst.ca/) was used to process and analyze the acquired raw data of peak intensities from the instrument [16]. Briefly, the raw data were pre-processed by eliminating features with >15% missing values, and the remaining missing values were replaced with limit of detection (LoD), which was set at 1/5 of the minimum positive value of each variable. Subsequently, the data were processed by normalization to the median, log transformation (base 10), and auto scaling (mean-centered and divided by the standard deviation of each variable). An array of statistical analyses, encompassing both univariate and multivariate analysis, was carried out. For univariate analysis, significance was determined by one-way ANOVA or independent samples t-test, and false discovery rate (FDR) adjusted P value < 0.05 was considered significant. For multivariate analysis, the supervised partial least squares discriminant analysis (PLS-DA) was performed to achieve enhanced group differentiation and gain deeper insights into the variables that contribute to categorization. The main method of screening differential metabolites between MDD and HC groups was based on the filtering criteria of false discovery rate (FDR) -adjusted P < 0.05 from two-sample t-tests, variable importance in projection (VIP) >1.5 from PLS-DA, and fold change (FC) >2.0 or < 0.5 for the absolute values of change between two group means [17, 18]. Moreover, univariate and multivariate ROC analyses were also performed using the biomarker analysis module of MetaboAnalyst web service [15]. The threshold value for Areas under the receiver–operator curves (AUC) was set at 0.5. An AUC closer to 1 indicates better performance of the test [19]. An AUC < 0.5 indicates a test that is not useful, 0.5–0.6 indicates a bad accuracy, 0.6–0.7 indicates a sufficient accuracy, 0.7–0.8 indicates a good accuracy, 0.8–0.9 indicates a very good accuracy, and more than 0.9 indicates excellent accuracy. The top 5, 10, 15, 25, 50, and 100 critical features were then used to build classification models which were validated on the 1/3 samples that were left out.

## Results

### Demographic and clinical characteristics of study subjects

A total of 107 adults diagnosed with MDD, consisting of 44 males and 63 females, were included in the study. Among them, 50 individuals were experiencing their first episode of MDD. Additionally, 97 HCs were recruited, including 28 males and 69 females. There were no significant differences in sex, education level, smokers, and drinkers between the two groups of participants (all *p*>0.05). However, it is worth noting that the age distribution in the MDD group is more varied compared to the HC group. The BMI of HC was significantly higher than MDD (23.71±3.07 vs. 22.44±4.46,*p*<0.001). Table 1 displays the clinical and demographic features of the participants.

**Table 1:** Basic information of the included participants

Variable	MDD (*n* = 107)	HC (*n* = 97)	*p*-value
Age, year, Mean ± SD	40.15 ± 15.53	40.33 ± 6.64	< 0.001
Sex (n, %)			0.067
Male	44 (41.1)	28 (28.9)	
Female	63 (58.9)	69 (71.1)	
Education level (n, %)			
Primary school	23 (21.5)	22 (22.7)	0.766
Secondary school	51 (47.7)	41 (42.3)	
High school	23 (21.5)	21 (21.6)	
Undergraduate or above	10 (9.3)	13 (13.4)	
Drinker (n, %)	11 (10.3)	16 (16.5)	0.191
Smoker (n, %)	8 (7.5)	15 (15.5)	0.072
BMI, kg/m2, Mean ± SD	22.44 ± 4.46	23.71 ± 3.07	< 0.001
First-episode (n, %)	50 (46.7)		
HAMD-24, Mean ± SD	26.98 ± 5.96	2.08 ± 1.63	< 0.001

*Abbreviations*: *BMI *Body mass index, *HAMD *Hamilton Depression Scale, *GAD *Generalized anxiety disorder, *SHAPS *Snaith-Hamilton Pleasure Scale

### Serum lipid profiles of individuals with MDD and HC

**Fig. 1:**
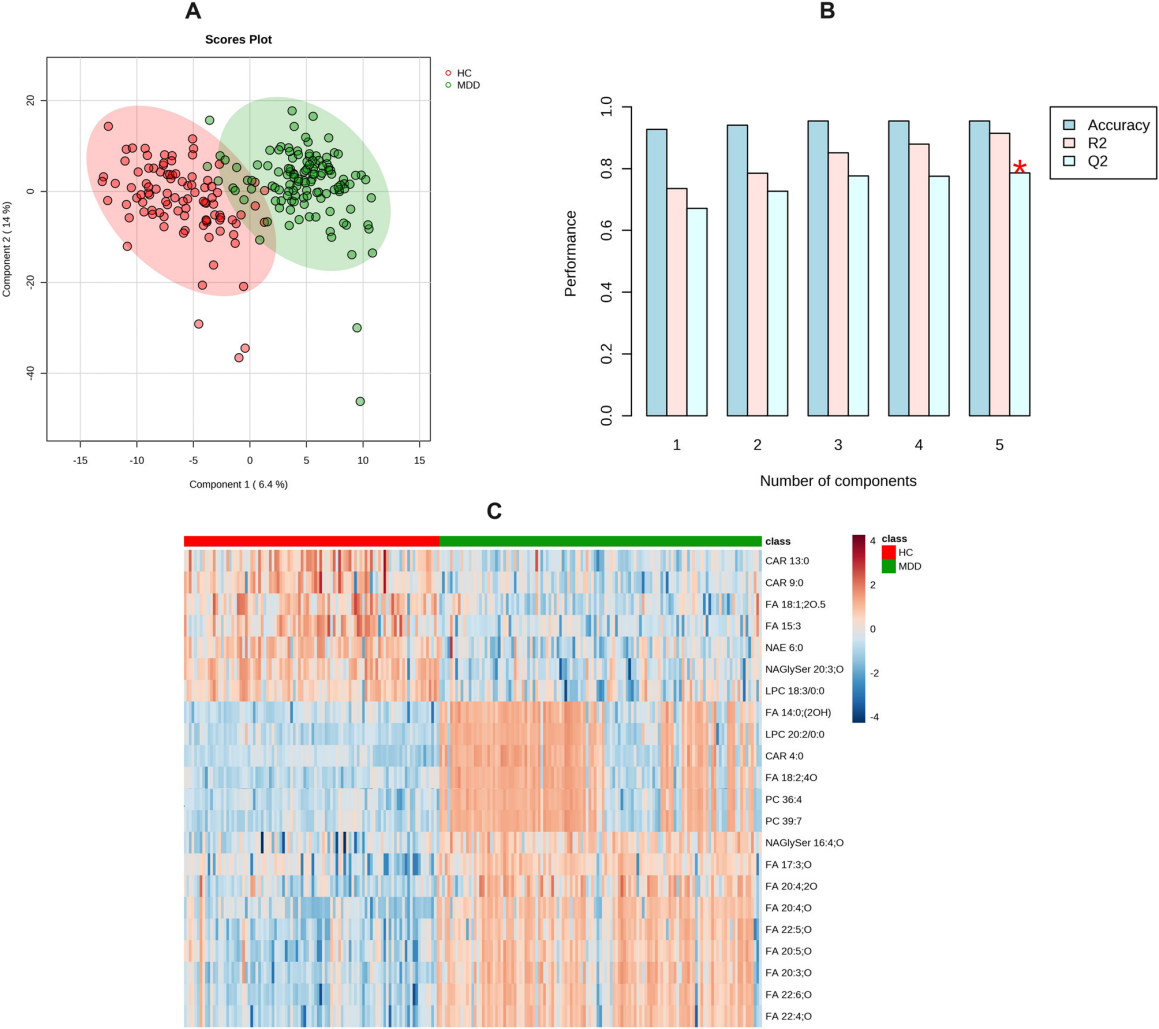
Lipidomic analysis of serum samples from MDD and HC groups. **A **2D PLS-DA scores plot of top 2 components. **B** Overview of the performance of the top 5 components. **C** The heatmap of representative lipids to distinguish MDD and HC

**Fig. 2:**
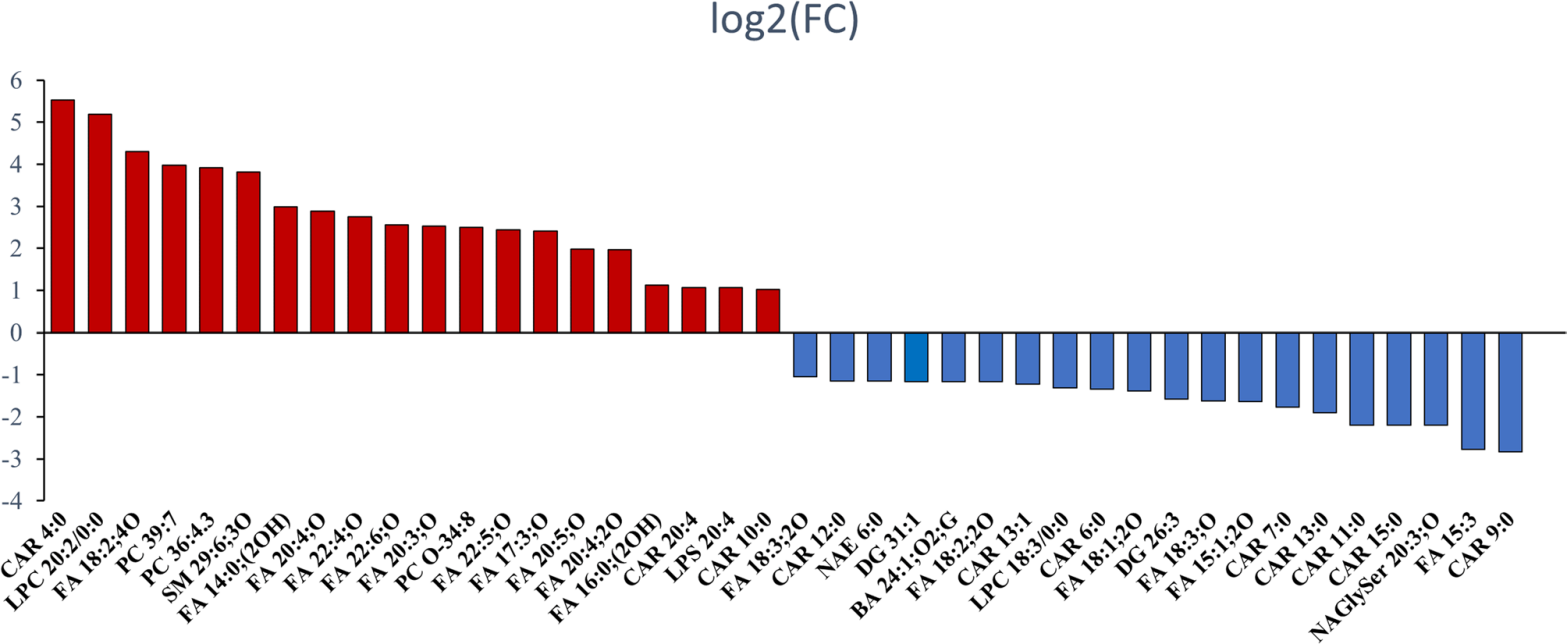
Total 40 differential lipids between MDD and HC groups. The abscissa represents each feature, the ordinate represents the fold change (FC) after the log2 transformation. Red: MDD > HC; Blue: MDD < HC. FC, fold change; FDR, false discovery rate; VIP, variable importance projection; CAR, acyl-carnitine; BA, bile acids; DG, Diglyceride; FA, fatty acid; LPS, Lipopolysaccharide; PC, phosphatidylcholine; SM, sphingomyelin; LPC, lysophosphatidylcholine; NAE, N-acylethanolamine; OxFA, oxidized fatty acid

For the untargeted lipidomic profiling, a total of 453 positive-mode features and 289 negative-mode features were identified in the MS/MS search and subsequently applied for MetaboAnalyst analysis (details of the 742 features are shown in the supplemental data file). Following the completion of data processing, a total of 694 features were incorporated into the data analysis to identify differences in lipidomic profiles between the MDD group and HC group. According to our principal components analysis (PCA) 3D scatter plot (Supplemental Figure 1A), the two comparison groups were well separated, especially by the second and third principal components (PCs). The scree plot indicated that the top five PCs accounted for 39.3% accumulated variance (Supplemental Figure 1B). The details of PLS-DA analysis are shown in Fig. 1, with remarkable separations of MDD vs. HCs. The lipids that differentiated between the groups were filtered using multivariate (i.e., PLS-DA) and univariate (i.e., fold change analysis and two-sample t-test) statistical significance criteria (VIP>1.5, FC>2.0 or <0.5, and FDR<0.05). The volcano plot is shown in Supplemental Figure 2. In total, significant differences in 40 identified lipids were reported between MDD and HC. Out of the total, 20 lipids showed a significant increase, whereas 20 lipids showed a significant decrease in individuals with MDD compared to HCs. Moreover, the majority of the discovered differential lipids consisted of oxidized fatty acids (OxFAs) and acyl-carnitines (CARs). The comprehensive information regarding these lipids can be found in Table 2. The log2 transformed FC for the 40 metabolites that show differential expression between individuals with MDD and HCs is presented in Fig. 2. The PLS-DA results of comparisons between the first-episode group and the relapse group, and the first-episode group with the control group, and relapse group with the control are shown in the Supplemental Figure 3. No eligible differential lipid was found between the first-episode group and the relapse group.

**Table 2:** Differentiating lipids between MDD and HC groups were identified from the lipidomic data

Lipids	FC	log_2_(FC)	FDR	VIP	Category
MDD > HC					
CAR 4:0	46.24	5.53	8.66E-21	2.91	CAR
CAR 20:4	2.10	1.07	5.98E-06	1.61	CAR
CAR 10:0	2.02	1.02	3.84E-07	1.78	CAR
LPC 20:2/0:0	36.43	5.19	1.88E-24	3.11	LPC
LPS 20:4	2.09	1.06	1.14E-10	2.16	LPS
FA 18:2;4O	19.70	4.30	2.05E-24	3.11	OxFA
FA 14:0;(2OH)	7.97	3.00	1.71E-22	3.01	OxFA
FA 20:4;O	7.41	2.89	7.36E-35	3.56	OxFA
FA 22:4;O	6.71	2.75	1.94E-26	3.21	OxFA
FA 22:6;O	5.87	2.55	1.44E-27	3.27	OxFA
FA 20:3;O	5.76	2.53	1.44E-27	3.27	OxFA
FA 22:5;O	5.43	2.44	3.47E-22	2.99	OxFA
FA 17:3;O	5.30	2.41	2.06E-15	2.56	OxFA
FA 20:5;O	3.98	1.99	8.11E-22	2.97	OxFA
FA 20:4;2O	3.93	1.97	9.72E-15	2.51	OxFA
FA 16:0;(2OH)	2.18	1.13	4.12E-14	2.46	OxFA
PC 39:7	15.75	3.98	3.13E-20	2.88	PC
PC 36:4	15.11	3.92	4.02E-21	2.93	PC
PC O-34:8	5.64	2.49	2.63E-12	2.31	PC
SM 29:6;3O	14.12	3.82	3.00E-12	2.31	SM
MDD < HC					
BA 24:1;O2;G	0.45	−1.16	8.52E-06	1.58	BASulfate
CAR 12:0	0.45	−1.14	5.91E-11	2.19	CAR
CAR 13:1	0.43	−1.22	2.33E-09	2.03	CAR
CAR 6:0	0.39	−1.34	2.65E-06	1.66	CAR
CAR 7:0	0.29	−1.78	3.19E-10	2.12	CAR
CAR 13:0	0.27	−1.89	1.17E-16	2.65	CAR
CAR 11:0	0.22	−2.19	1.06E-09	2.07	CAR
CAR 15:0	0.22	−2.19	1.11E-09	2.06	CAR
CAR 9:0	0.14	−2.83	9.59E-16	2.59	CAR
DG 26:3	0.34	−1.58	1.16E-11	2.25	DG
DG 31:1	0.45	−1.16	1.64E-07	1.82	DG
FA 15:3	0.15	−2.78	9.59E-16	2.59	FA
LPC 18:3/0:0	0.40	−1.31	3.67E-14	2.47	LPC
NAE 6:0	0.45	−1.15	8.64E-17	2.66	NAE
NAGlySer 20:3;O	0.22	−2.20	3.37E-20	2.87	NAGlySer
FA 18:3;2O	0.48	−1.05	3.30E-06	1.65	OxFA
FA 18:2;2O	0.44	−1.17	7.82E-12	2.27	OxFA
FA 18:1;2O	0.38	−1.38	2.53E-17	2.70	OxFA
FA 18:3;O	0.32	−1.63	4.69E-14	2.46	OxFA
FA 15:1;2O	0.32	−1.63	1.04E-06	1.72	OxFA

*Abbreviations*: *FC *Fold change, *FDR *False discovery rate, *VIP *Variable importance projection, *CAR *Acyl-carnitine, *BA *Bile acids, *DG *Diglyceride, *FA *Fatty acid, *LPS *Lipopolysaccharide, *PC *Phosphatidylcholine, *SM * Sphingomyelin, *LPC *Lysophosphatidylcholine, *NAE *N-acylethanolamine, *OxFA *Oxidized fatty acid

We further explored the correlations between the basic characteristics and the identified differential metabolites in the MDD and HC groups. The results revealed that the majority of metabolites elevated in the MDD group exhibited positive correlations with HAMD scores and negative correlations with BMI. Conversely, a multitude of metabolites with decreased levels in the MDD group demonstrated negative correlations with both HAMD scores and age, while showing positive correlations with BMI. Details are shown in Supplemental Figure 4.

### ROC analysis revealed candidate lipid biomarkers for MDD

**Fig. 3:**
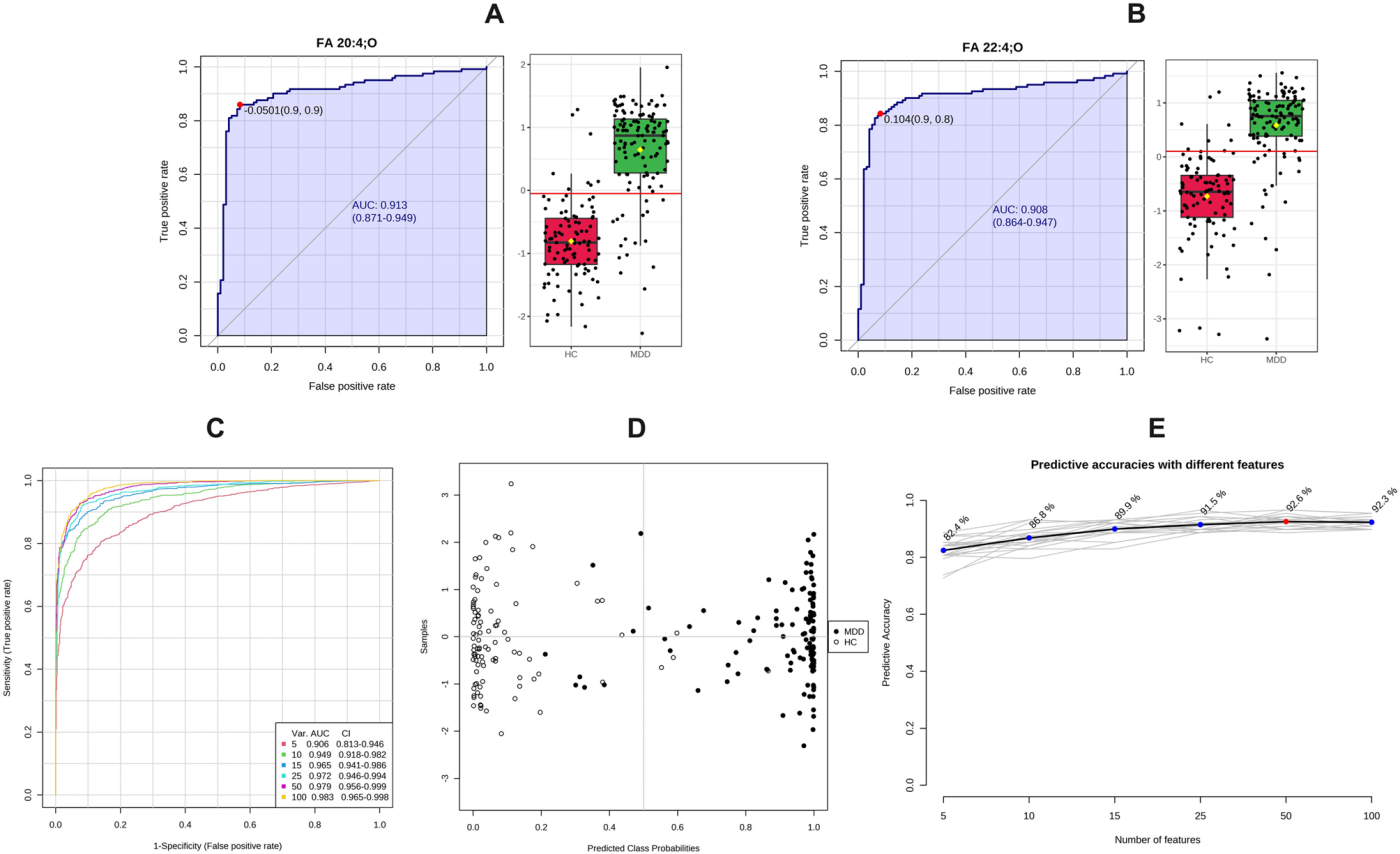
ROC analysis revealed candidate lipid biomarkers for MDD diagnosis. **A** and **B** ROCs of FA 20:4;O, and FA 22:4;O. The left side of each figure represents the ROC for differentiating the MDD group from the HC group, and the boxplots on the right side are the feature intensities of the two groups, y-axis refers to the relative value after normalization of the peak intensities. **C** Multivariate ROC constructed with 2-100 lipids based on the cross-validation (CV) performance. Each curve represents the potential of the top 5, 10, 15, 25, 50, and 100 features in differentiating the MDD group from the HC group. **D** The predicted class probabilities (average of the cross-validation) for each sample using the 5 features model of lipids. **E** The corresponding predictive accuracy of each PLS-DA model is constructed with different numbers of features. The predictive accuracy of 5 to 100 features is from 82.4–92.3%, respectively

To estimate the diagnostic value of the dysregulated lipids, ROC analyses were conducted on each plasma metabolite. Two OxFAs were identified as the most efficient diagnostic biomarkers for MDD from HCs, with an AUC of 0.913 and 0.908, respectively (see Fig. 3A and B). The ROCs of 25 lipids provided good classification ability for MDD from HCs, with AUC>0.8 (Supplemental Table 1). For multivariate ROCs, the top 5, 10, 15, 25, 50, and 100 lipids were used to build classification models with two-thirds samples, then were validated on the remaining one-third of samples. These curves attained AUCs exceeding 0.983 for the top 100 lipids (Fig. 3C). The corresponding predictive accuracy of each PLS-DA model constructed with different numbers of features is shown in Fig. 3D and E.

## Discussion

The foregoing studies have emphasized the potential significance of peripheral lipids, such as cholesterol and polyunsaturated fatty acids (PUFAs), in predicting, categorizing, and treating MDD [20]. Our study emphasized the widespread abnormalities of two types of lipids (i.e., OxFAs and CARs) that may be associated with inflammation in patients with depression. The primary findings were as follows: (1) Patients with MDD showed an increase in 11 types of OxFA in patients with depression (mainly belonging to 22 and 24 carbon chain lengths), and a decrease in 5 types of OxFA (mainly with 18 carbon chain lengths); (2) In MDD, eight types of CARs decreased (mainly singular carbon chain structures) and 3 types of CARs increased (all numerical carbon chain structures); (3) Lipid profiles analysis could provide a high ROC level for identifying depression, primarily attributed to OxFAs.

OxFAs refer to the compounds that result from the oxidation of fatty acids. The process of fatty acid oxidation is initiated on the outer mitochondrial membrane. The existing evidence pointed out that polyunsaturated fatty acids (PUFAs), specifically those with 18, 20, and 22 carbons, can easily be autooxidized in mammals [21]. The decreased levels of PUFAs (especially n-3 PUFAs) in MDD have been reported in multiple studies [22, 23]. The preceding research indicates that a lack of PUFAs may result in the disruption of serotonin and dopamine control, oxidative stress, chronic inflammation, and abnormalities in the structure and function of the cortex, further leading to mood dysregulation [24]. Furthermore, a growing body of research has demonstrated the positive effects of PUFA supplementation in alleviating depressive symptoms in individuals with MDD [25, 26]. Although our non-targeted lipidomic profiles did not identify the difference in PUFA between MDD and HC groups, we found that most of the detected OxFAs were higher in individuals with MDD than in the control group, especially OxFAs with 20 and 22 carbons. Previous literature has provided minimal information on the direct mechanism linking the rise of OxFAs with depression. A recent study reported the elevated levels of OxFAs negatively impact antioxidant activity of high-density lipoprotein (HDL) in diabetes [27]. Moreover, the oxidized products (such as, OxFAs) from PUFA may also induce apoptosis in cultured cells [28]. The higher levels of OxFAs in atherosclerosis, fatty liver, and active rheumatoid arthritis have also been widely and consistently reported [29–31]. The increased oxidative stress contributes to the formation of certain oxidized lipids [32, 33]. We reported a decrease in a fatty acid (i.e., FA 15:3), which further corroborates the aforementioned perspective.

In this study, we observed notable changes in CARs (with 8 decreases and 3 increases) in MDD. CARs have a crucial function in sustaining energy metabolism and facilitating elimination of oxidative products by transporting fatty acids into the mitochondria for beta oxidation [34, 35]. Consistent with our findings, most previous studies have reported a decrease in CARs in patients with MDD [36–38]. The previously documented putative mechanisms include neuroplasticity effect, oxidative stress and neurotransmitter regulation [39, 40]. Due to the unique neuroprotective effects of CARs, which include anti-inflammatory and antioxidant benefits [41], clinical research has shown that supplementing with free carnitine and acetyl-1-carnitine could significantly reduce depressive symptoms compared to placebo/no intervention [42, 43]. In our current study, most of the identified CARs were medium- and long-chain. Evidence from two large databases suggests they may be intermediates in mitochondrial fatty acid β-oxidation, indicating metabolic inflexibility or mitochondrial dysfunction in both major depression and depressive mood [44, 45]. Additionally, it should be noted that there were also three elevated levels of CARs (i.e., CAR 4:0, CAR 10:0, and CAR 20:4) in the MDD group. The existing evidence from a review article indicated that increases in CARs may lead to a decrease in the activities of depressed rats [46, 47]. Given the conflicting results presented above, our findings require further validation, and clinicians should also be very cautious about supplementing therapy with CARs.

For other lipids, we also found sporadic differences between MDD and HC groups. The current study indicated an increase in 3 types of PCs and 1 type of SM, as well as a decrease in 2 types of DGs, and a decrease in NAE. PCs and SMs are predominantly maintained on the outer leaflet [48], they have similarities and specific interactions. However, the exact mechanism in depressed patients remains unclear. A recent study pointed out that PC and SM are also elevated in type 2 diabetic kidney disease, which may be involved in abnormal phospholipids activation of the sorbitol pathway, oxidative stress, and activation of protein kinase C [49]. There were a small number of other types of lipids that also differed between MDD and HC groups, but only one or two of each type, making it difficult to figure out their characteristics and patterns of change. Their changes and rules should be further explored from targeted lipidomics in future studies.

Although our study provides new evidence for the lipidomics of MDD, the limitations of the study still need to be addressed, and the results should be interpreted with caution. Firstly, similar to other cross-sectional studies, it is difficult for the study to explain causal relationships. Secondly, our study did not investigate the nutritional supplements, dietary status, as well as intake of lipid-lowering drugs (e.g. statins) of the study subjects, which could potentially influence the outcomes. Moreover, our current study focused on patients with MDD as their primary diagnosis. We did not rule out the subjects who might have other health conditions, such as anxiety, or insomnia, which may also have an impact on the results. Additionally, due to the high heterogeneity of untargeted lipidomics research [50], our findings may relate to the selection, the preprocessing methods and test conditions of our samples, or the data preprocessing method we selected. The main reported alterations of our study (acyl-carnitines, oxidized free fatty acids, lysophospholipids) represented lipids that would be most sensitive to subtle biases in sample processing. It was reported that even a small difference in storage time at room temperature before centrifugation could lead to substantial changes in the levels of certain lipid groups [51]. Thus, our results may not be reproducible in future studies. Finally, larger samples and more standardized lipidomics testing conditions are needed to justify the findings of this study. Matching of basic variables is also an important aspect that future research should focus on to enhance the reproducibility of the results.

## Conclusions

In summary, our study reported a wide range of lipid differences, particularly increased OxFAs and decreased CARs, between individuals with MDD and HCs. Furthermore, some OxFAs exhibited promising diagnostic capabilities for MDD. Supplementation with PUFAs and acyl-carnitines warrants further investigation as a potential strategy for the management of MDD. Nevertheless, it is necessary to conduct further investigation and exercise caution while considering their various types and proportions.

## Supplementary Information

Supplementary Material 1.

Supplementary Material 2.

Supplementary Material 3.

Supplementary Material 4.

## Data Availability

The datasets used or analyzed during the current study are available from the corresponding author upon reasonable request.

## Abbreviations

OxFA: Oxidized fatty acid
CAR: Acyl-carnitine
MDD: Major depressive disorder
HC: Healthy controls
FDR: False discovery rate
VIP: Variable importance in projection
FC: Fold change
ROC: Receiver operating characteristic
LPC: Lysophosphatidylcholine
LPE: Lysophosphatidylethanolamine
PE: Phosphatidylethanolamine
PC: Phosphatidylcholine
SM: Sphingomyelin
DSM-V: Diagnostic and Statistical Manual of Mental Disorders-5th version
MDE: Major depressive episode
HAMD: Hamilton Depression Scale
QC: Quality control
DDA: Data-dependent acquisition
SD: Standard deviation
IQR: Interquartile range
LoD: Limit of Detection
PLS-DA: Partial least squares discriminant analysis
AUC: Areas under the receiver–operator curves
PUFA: Polyunsaturated fatty acid
