# Supplementary material for: Lipidomic analyses reveal the dysregulation of oxidized fatty acids (OxFAs) and acyl-carnitines (CARs) in major depressive disorder: a case-control study
Source: BMC Psychiatry. 2025 Aug 1;25:752. doi: 10.1186/s12888-025-07191-7 (PMC12317606; doi:10.1186/s12888-025-07191-7)
Supplement: Supplementary file 2 — Supplementary Material 2. [file 12888_2025_7191_MOESM2_ESM.docx]

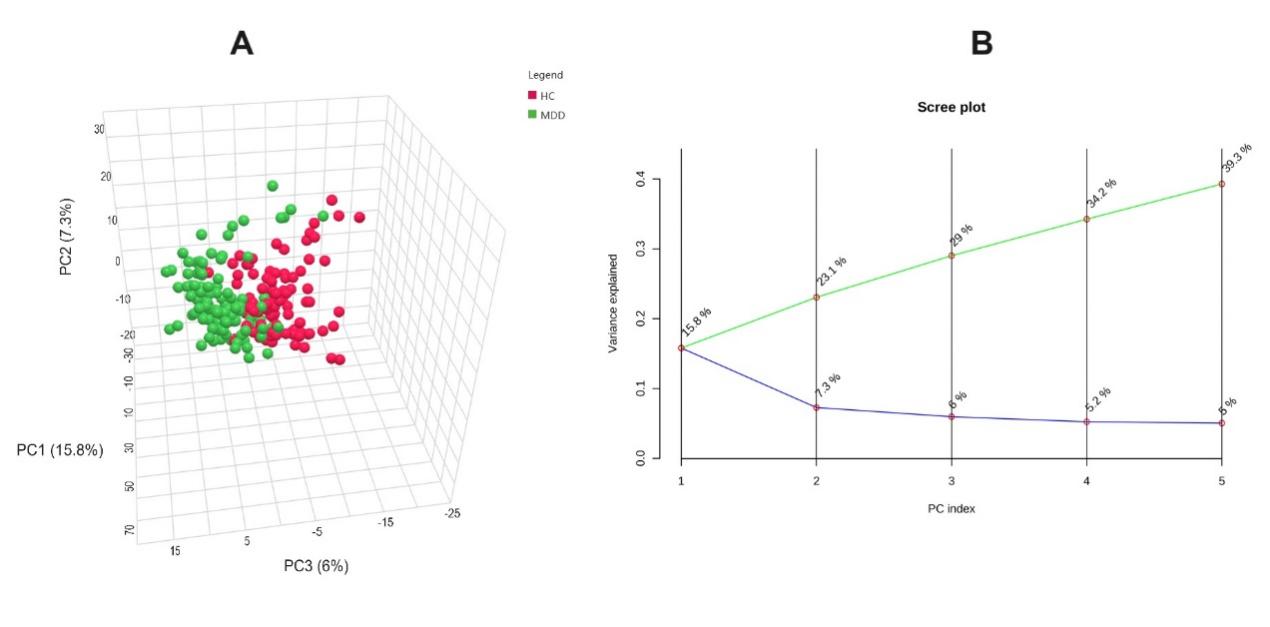


**Supplemental Figure 1.** Overview of PCA results for MDD and HC. (A) Top 3 components of PCA for untargeted lipid profiling of serum samples in MDD, and HCsamples. (B) Scree plot of top 5 components.


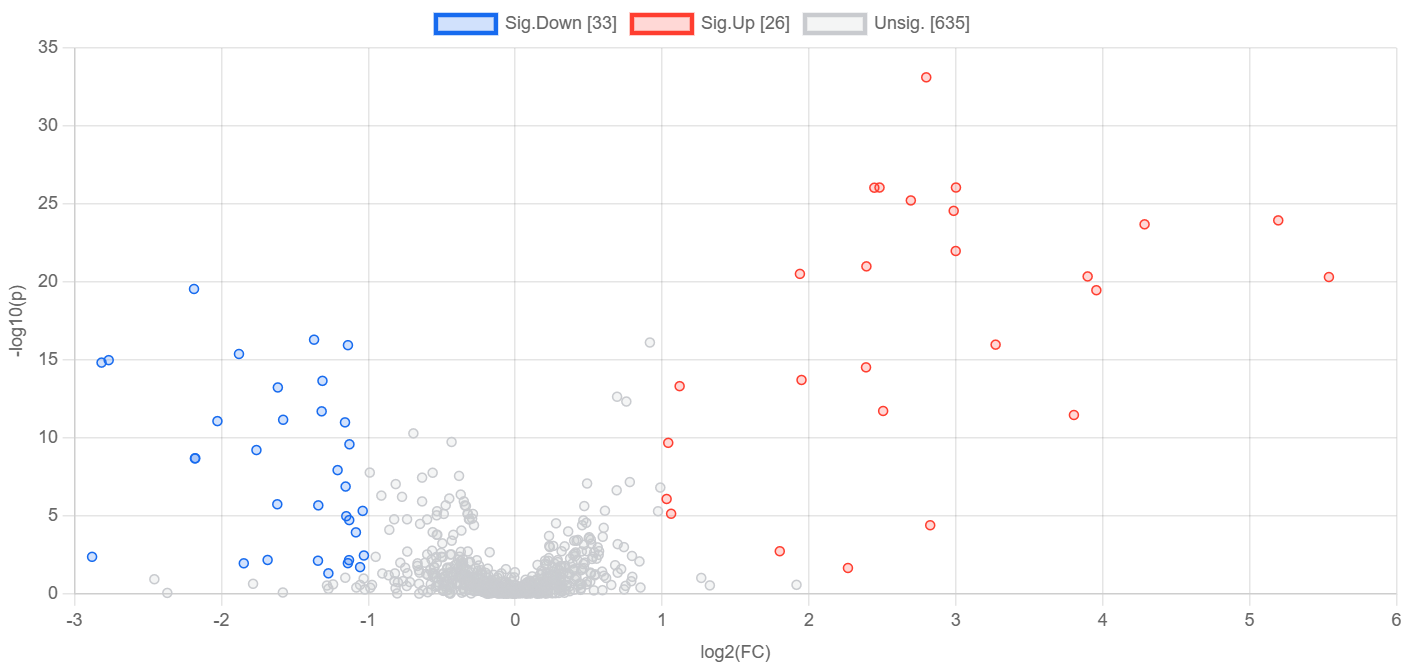


**Supplemental Figure 2.** Volcano plot of features with false discovery rate (FDR) < 0.05 and |FC| > 2 from univariate analysis. Red-colored dots denote up-regulated in MDD, blue-colored dots denote down-regulated in MDD, and grey-colored dots denote metabolites with non-significant change.


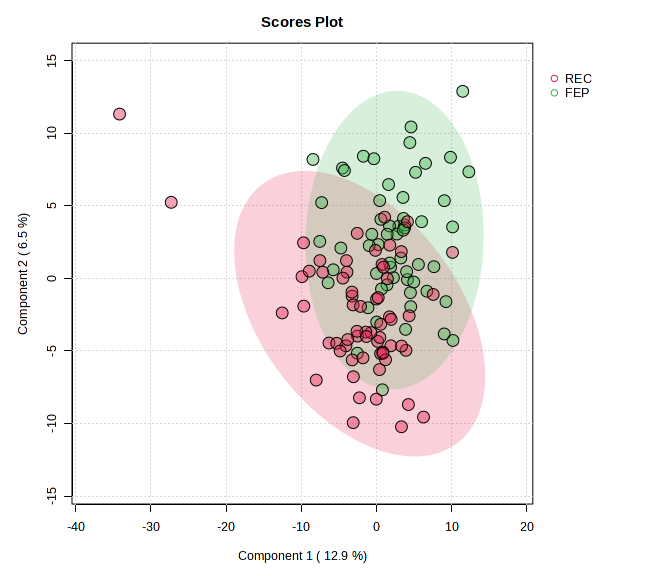

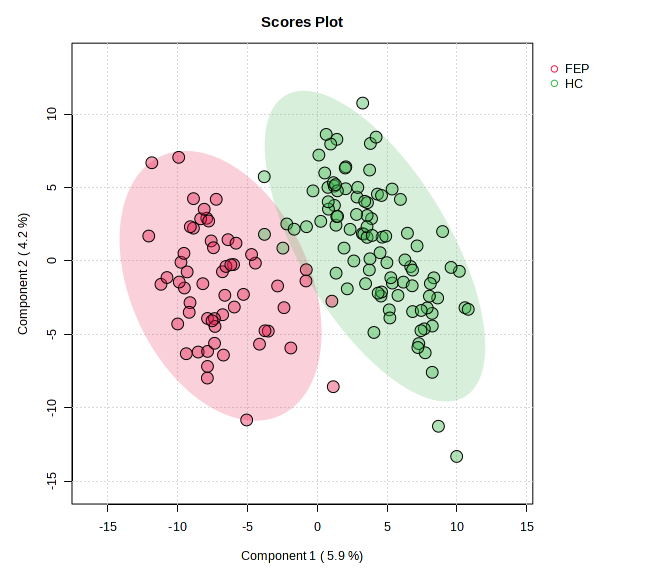

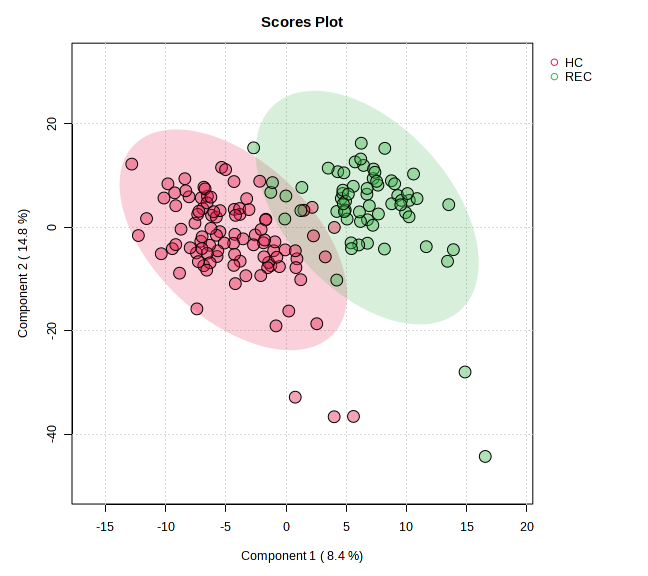


**A B C**

**Supplemental Figure 3.** 2D PLS-DA scores plot of top 2 components. (A) first-episode group vs. relapse group, (B) first-episode group vs. control group, (C) relapse group vs. control group. FEP, First-episode; REC, Recurrence; HC, healthy control.

**Supplemental Figure 4.** Spearman correlation analysis results of Basic information and identified differential metabolites in MDD and HC. The metabolites above the bold solid line were elevated in the MDD group, while those below were decreased..

**Supplemental Table 1. Areas under the receiver-operating characteristic curves (AUCs) of lipids**

| **Lipids** | **AUC** | **P-value** | **FC** |
| --- | --- | --- | --- |
| FA 20:4;O | 0.913 | 1.10E-36 | 2.77 |
| FA 22:4;O | 0.908 | 4.30E-28 | 2.63 |
| FA 22:6;O | 0.899 | 3.30E-29 | 2.41 |
| FA 20:3;O | 0.886 | 5.25E-29 | 2.33 |
| NAGlySer 20:3;O | 0.868 | 5.72E-22 | -2.27 |
| FA 22:5;O | 0.866 | 1.45E-23 | 2.3 |
| FA 17:3;O | 0.859 | 9.98E-17 | 2.29 |
| FA 20:5;O | 0.859 | 4.84E-23 | 1.75 |
| LPC 20:2/0:0 | 0.841 | 1.14E-26 | 5.3 |
| FA 18:2;4O | 0.84 | 2.33E-26 | 4.32 |
| CAR 9:0 | 0.839 | 4.73E-17 | -2.86 |
| NAGlySer 16:4;O | 0.838 | 1.89E-18 | 0.87 |
| FA 14:0;(2OH) | 0.836 | 1.35E-24 | 2.91 |
| NAE 6:0 | 0.832 | 3.12E-18 | -1.19 |
| FA 18:1;2O | 0.831 | 1.17E-18 | -1.47 |
| LPC 13:0 | 0.828 | 1.96E-14 | 0.69 |
| CAR 13:0 | 0.824 | 1.21E-17 | -1.95 |
| FA 16:0;(2OH) | 0.824 | 1.81E-15 | 1.06 |
| LPA 20:4 | 0.816 | 9.30E-15 | 0.62 |
| FA 18:3;O | 0.816 | 2.28E-15 | -1.72 |
| LPC 18:3/0:0 | 0.815 | 7.96E-16 | -1.34 |
| PC 36:4 | 0.81 | 7.70E-23 | 3.96 |
| FA 15:3 | 0.809 | 3.15E-17 | -3.02 |
| PC 39:7 | 0.808 | 7.36E-22 | 4.06 |
| FA 20:4;2O | 0.802 | 6.71E-16 | 1.78 |
