# Supplementary material for: Lipidomic analyses reveal the dysregulation of oxidized fatty acids (OxFAs) and acyl-carnitines (CARs) in major depressive disorder: a case-control study
Source: BMC Psychiatry. 2025 Aug 1;25:752. doi: 10.1186/s12888-025-07191-7 (PMC12317606; doi:10.1186/s12888-025-07191-7)
Supplement: Supplementary file 3 — Supplementary Material 3. [file 12888_2025_7191_MOESM3_ESM.pdf]

驻马店市精神病医院伦理委员会伦理审查批件

批件号：2021-006-02

研究题目：重性抑郁障碍炎症代谢特征及机制研究
研究所属专业：精神病
研究目的：探究重症抑郁障碍患者认知障碍的炎症代谢机制及相关健康危险因素
申办者的名称：驻马店市精神病医院（驻马店市第二人民医院）<br>地址：河南省驻马店市雪松路东段51号
主要研究者：何磊
审查方式：快速审查
审查类别：复审
审查日期：2021年09月22日
审查地点：驻马店市精神病医院门诊楼五楼会议室
联系人及联系电话：李小新　　13193716801
**本伦理委员会已经审阅了以下文件：**<br>1. 复审申请表<br>2. 主要研究人员履历<br>3. 修正的试验方案（版本号：2.0 版本日期：2021.09.16）<br>4. 修正的知情同意书（版本号：2.0 版本日期：2021.09.16）<br>5. 修正的病例报告表（版本号：2.0 版本日期：2021.09.16）<br>6. 修正的研究者操作手册（版本号：2.0 版本日期：2021.09.16）<br>7. 主要研究者利益冲突声明<br>8. 学术委员会审查意见
到会情况：实到：　人；缺席：　人；回避：　人<br>投票情况：<br>1. 知情同意书：同意：　票；作必要的修正后同意：　票；作必要的修正后重审：　票；不同意：　票；终止或暂停已批准的研究：　票<br>2. 研究方案：　同意：　票；作必要的修正后同意：　票；作必要的修正后重审：　票；不同意：　票；终止或暂停已批准的研究：　票

扫描全能王

总体意见	同意
跟踪审查频率	12 个月

审批意见：

☑ 同意

☐ 作必要的修正后同意

☐ 作必要的修正后重审

☐ 不同意

☐ 终止或暂停已批准的研究

备注：

说明：本伦理委员会批件自审批日起，有效期壹年。

附件：伦理委员会声明及伦理委员会委员名单

伦理委员会主任签名：[illegible]

伦理委员会公章 驻马店市精神病医院伦理委员会

审批日期：2021年9月22日

CS 扫描全能王

附件

伦理委员会声明

本伦理委员会的职责、人员组成、操作程序及记录遵循 ICH-GCP、中国的相关法律和法规。

This ethics committee's responsibilities, composition, function, operations and records are fully compliant with ICH-GCP, and related regulation and law of China.

伦理委员会委员名单				
姓　名	性　别	任　职	工作单位	职称职务
贾金鼎	男	主任委员	驻马店市精神病医院	精神科主任医师<br>纪检书记
范大庆	男	副主任委员	驻马店市精神病医院	精神科主任医师<br>工会主席
张　翼	男	委员	驻马店市精神病医院	高级会计师<br>副院长
张清华	男	委员	驻马店市精神病医院	神经内科主任医师<br>科主任
朱玉星	女	委员	驻马店市精神病医院	主任护师<br>科主任
李小新	女	委员	驻马店市精神病医院	主任药师<br>科主任
栾　琴	女	委员	驻马店市精神病医院	副主任医师<br>科主任
刘　飞	男	委员	河南发时达律师事务所	律师
张爱香	女	委员	社区居民	无

本伦理委员会委员名单自 2020 年 5 月 6 日起生效，有效期 3 年。

驻马店市精神病医院伦理委员会

驻马店市精神病医院伦理委员会

CS 扫描全能王
