# Supplementary material for: Lipidomic analyses reveal the dysregulation of oxidized fatty acids (OxFAs) and acyl-carnitines (CARs) in major depressive disorder: a case-control study
Source: BMC Psychiatry. 2025 Aug 1;25:752. doi: 10.1186/s12888-025-07191-7 (PMC12317606; doi:10.1186/s12888-025-07191-7)
Supplement: Supplementary file 4 — Supplementary Material 4. [file 12888_2025_7191_MOESM4_ESM.docx]

Approval No.: 2021-006-02

Research Title: Study on Inflammatory and Metabolic Characteristics and Mechanisms in Major Depressive Disorder

Research Specialty: Psychiatry

Research Objective: To explore the inflammatory and metabolic mechanisms of cognitive impairment and related health risk factors in patients with Major Depressive Disorder.

Sponsor's Name: Zhumadian Psychiatric Hospital (Zhumadian Second People's Hospital)
Address: No. 51, East Section of Xuesong Road, Zhumadian City, Henan Province

Principal Investigator: He Lei

Review Method: Expedited Review

Review Category: Re-review

Review Date: September 22, 2021

Review Location: Conference Room, 5th Floor, Outpatient Building, Zhumadian Psychiatric Hospital
Contact Person & Phone Number: Li Xiaoxin 13193716801

This Ethics Committee has reviewed the following documents:

Re-review Application Form

Curriculum Vitae of Principal Investigator

Revised Study Protocol (Version No.: 2.0, Version Date: 2021.09.16)

Revised Informed Consent Form (Version No.: 2.0, Version Date: 2021.09.16)

Revised Case Report Form (Version No.: 2.0, Version Date: 2021.09.16)

Revised Investigator Brochure (Version No.: 2.0, Version Date: 2021.09.16)

Principal Investigator Conflict of Interest Statement

Academic Committee Review Opinion

Attendance: Present: [Number] persons; Absent: [Number] persons; Recused: [Number] persons
Voting Status:

Informed Consent Form: Approve: [Number] votes; Approve after necessary revisions: [Number] votes; Re-review after necessary revisions: [Number] votes; Disapprove: [Number] votes; Terminate or suspend approved research: [Number] votes

Study Protocol: Approve: [Number] votes; Approve after necessary revisions: [Number] votes; Re-review after necessary revisions: [Number] votes; Disapprove: [Number] votes; Terminate or suspend approved research: [Number] votes

===== Page 2 =====

Overall Opinion: Approve
Follow-up Review Frequency: 12 months

Approval Opinion:
☑ Approve
□ Approve after necessary revisions
□ Re-review after necessary revisions
□ Disapprove
□ Terminate or suspend approved research

Remarks: [Blank]

Note: This Ethics Committee approval document is valid for one year from the date of approval.
Attachment: Ethics Committee Statement and List of Ethics Committee Members

Signature of Ethics Committee Chair:
Seal of Ethics Committee
Approval Date: September 30, 2021

===== Page 3 =====

Attachment

****Ethics Committee Statement****

The responsibilities, composition, operating procedures, and records of this Ethics Committee comply with ICH-GCP, and relevant Chinese laws and regulations.

This ethics committee’s responsibilities, composition, function, operations and records are fully compliant with ICH-GCP, and related regulation and law of China.

**List of Ethics Committee Members**

| Name | Gender | Position | Affiliation | Title/Position |
| --- | --- | --- | --- | --- |
| :----------- | :----- | :------------------ | :--------------------------- | :----------------------------------------- |
| Jia Jinding | Male | Chairperson | Zhumadian Psychiatric Hospital | Chief Physician, Discipline Inspection Secretary |
| Fan Daqing | Male | Vice-Chairperson | Zhumadian Psychiatric Hospital | Chief Physician, Trade Union Chairman |
| Zhang Yi | Male | Member | Zhumadian Psychiatric Hospital | Senior Accountant, Vice President |
| Zhang Qinghua | Male | Member | Zhumadian Psychiatric Hospital | Chief Physician (Neurology), Department Head |
| Zhu Yuxing | Female | Member | Zhumadian Psychiatric Hospital | Chief Nurse, Department Head |
| Li Xiaoxin | Female | Member | Zhumadian Psychiatric Hospital | Chief Pharmacist, Department Head |
| Luan Qin | Female | Member | Zhumadian Psychiatric Hospital | Associate Chief Physician, Department Head |
| Liu Fei | Male | Member | Henan Fashida Law Firm | Lawyer |
| Zhang Aixiang | Female | Member | Community Resident | None |

This list of Ethics Committee Members is effective from May 6, 2020, and is valid for 3 years.

Ethics Committee of Zhumadian Psychiatric Hospital
